# Designing a Single Lithium Disilicate Crown: A Computer-Aided Design and Computer-Aided Manufacturing Approach With Exocad Software

**DOI:** 10.7759/cureus.57384

**Published:** 2024-04-01

**Authors:** Jahnavi P Gorripati, Surekha A Godbole Dubey

**Affiliations:** 1 Department of Prosthodontics, Sharad Pawar Dental College and Hospital, Datta Meghe Institute of Higher Education and Research, Wardha, IND

**Keywords:** ips e.max cad, exocad designing, anterior esthetic, lithium disilicate, cadcam

## Abstract

This case report presents an overview of the workflow and advantages of computer-aided design and manufacture (CAD/CAM) technology in designing single crowns for anterior restoration. The workflow of CAD/CAM systems involves extraoral scanning, virtual crown design, and automated milling processes. The advantages of CAD/CAM technology include enhanced accuracy, reduced chairside time, and improved patient satisfaction. Considerations for material selection, clinical indications, and associated factors in CAD/CAM single-crown design are also discussed. Additionally, CAD/CAM provided accurate and lifelike restoration contours, ensuring optimal fit, function, and aesthetics. This technology proved highly beneficial in this case for several reasons. This case illustrates the significant advantages of CAD/CAM technology in modern dental practice, offering precise, efficient, and aesthetically pleasing solutions for single dental crowns.

## Introduction

Computer-aided design and manufacture (CAD/CAM) technologies have revolutionized prosthetic dentistry in numerous ways, such as enhancing treatment options for various dental indications, such as anterior and posterior restoration, including three-unit or more fixed partial dentures (FPD), complete dentures, thin veneers, and crowns. This versatility allows dentists to choose the most suitable restorative solution based on the specific needs of each case [1]. Diverse restorative materials in these systems support the use of various materials, including zirconia/all-ceramics, metals, and composite materials. This flexibility enables clinicians to select the most appropriate material that balances aesthetics, strength, and durability for each case [2]. There are both chairside and laboratory applications in which chairside systems allow dentists to fabricate restorations directly in the office during a single visit, offering convenience and time efficiency for patients. Laboratory-based systems provide dental technicians with advanced tools to design and manufacture high-quality restorations with meticulous detail and precision [3].

In aesthetic and functional restorations, CAD/CAM technology enables the creation of highly aesthetic and strong restorations that closely resemble natural teeth. The digital design process allows for precise customization of restoration shapes and contours to achieve optimal aesthetic results while ensuring proper function and occlusion [4]. The integration of CAD/CAM technology in restorative dentistry often results in improved patient experiences. Chairside CAD/CAM systems offer same-day restorations, eliminating the need for multiple appointments and temporary restorations. Additionally, the digital workflow minimizes the discomfort associated with traditional impression-taking methods, enhancing overall patient comfort and satisfaction [5].

Modern CAD/CAM systems feature advanced software for precise tool path control and restorative design as well as improved scanning technologies that enhance the detection of delicate margins [6]. Furthermore, there have been significant advancements in material science in recent years. This case report outlines the process undertaken to create a solitary lithium disilicate crown using Exocad software through CAD/CAM.

## Case presentation

**Figure 1:**
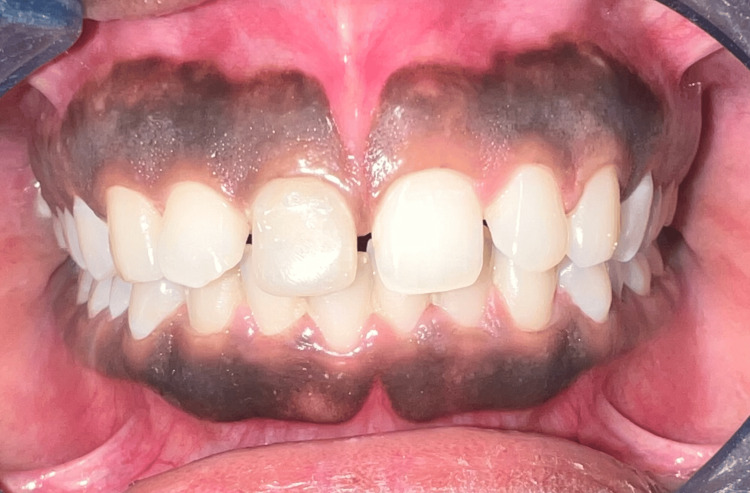
Preoperative intraoral picture

A female patient, aged 23, came to the Department of Prosthodontics at Sharad Pawar Dental College and sought dental consultation after receiving recent endodontic treatment on tooth #11. She expressed dissatisfaction with the appearance of the tooth (Figure 1). In addition to this, midline diastema was observed due to a high frenal attachment. Consequently, the patient was advised to initiate a comprehensive treatment plan, such as a frenectomy followed by an orthodontic closure of the space, despite the patient's request for treatment to be limited to tooth #11 only.

**Figure 2:**
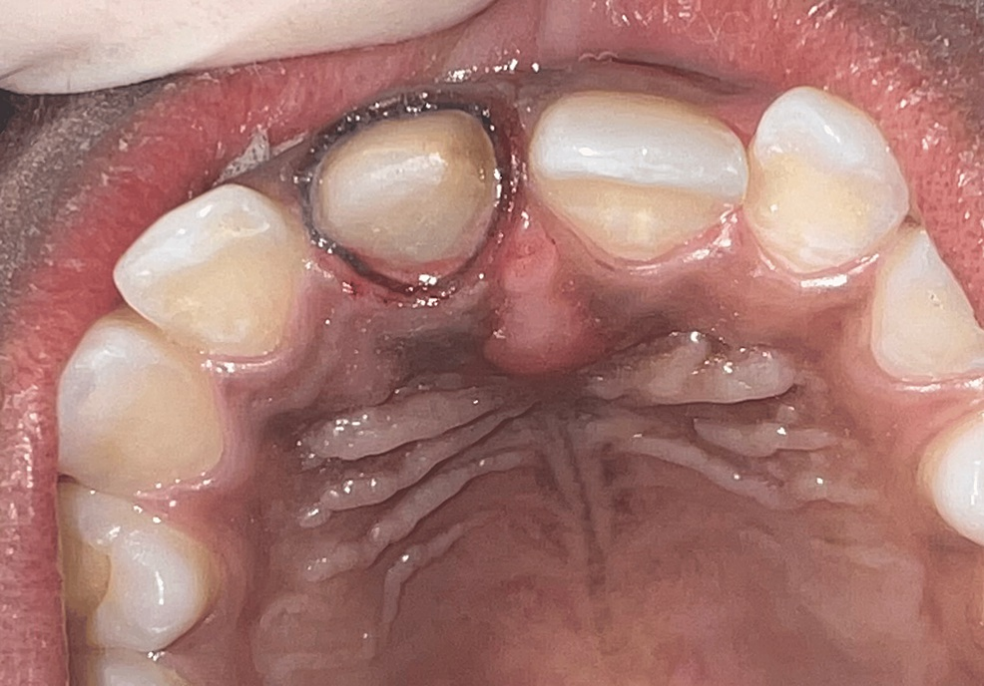
Tooth preparation of the maxillary right central incisor with gingival retraction cord

To address the patient's aesthetic concerns regarding tooth #11, a high-translucent and strength e.max crown was fabricated using CAD/CAM technology. This crown was milled to a thickness as thin as 300 µm axially, creating a contact lens effect on tooth #11's gingival margin on labial surface. After a thorough examination, the preparation process for the restoration of tooth #11 was initiated (Figure 2). The next step involved making an impression using polyvinyl siloxane with both putty and light body consistency. These impressions were then used to produce casts, which were scanned using an extraoral scanner (inEos X5, Dentsply Sirona). Subsequently, the resulting STL data was transferred to the Exocad design software.

**Figure 3:**
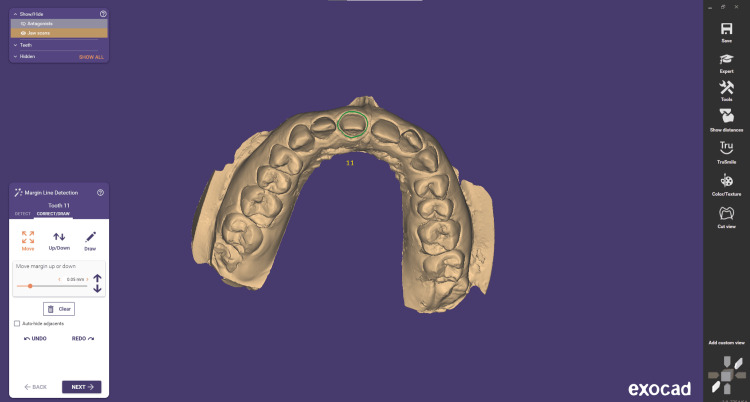
Margin line detection and manipulation of the margin

Step 1 is margin detection, and the initial step in crown design is to define the margin for the prepared teeth. This is done by positioning the initial seed point, which serves as the starting point for the automatic detection of the margin line (Figure 3).

**Figure 4:**
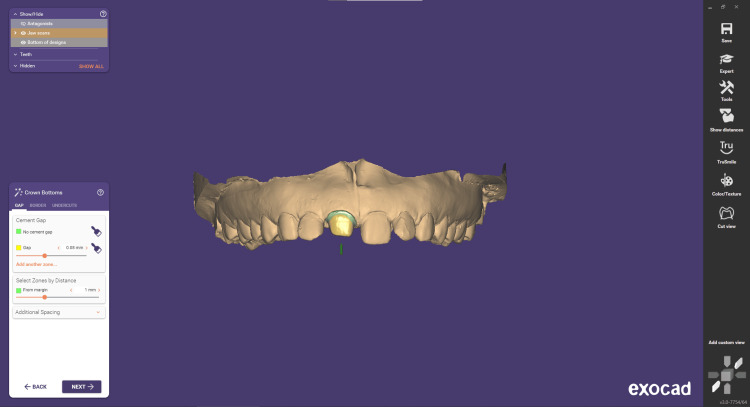
Crown insertion direction

Step 2 is correct/draw. At this stage, users can manipulate individual control points by dragging and adjusting them as needed. They can add or remove points as required and refine the margin line to ensure precise customization according to the specific requirements (Figure 3). Step 3 is insertion direction. In many cases, the software can accurately detect the proper insertion axis without requiring manual input. Therefore, users may not always be prompted to specify the insertion axis during the CAD design process (Figure 4).

**Figure 5:**
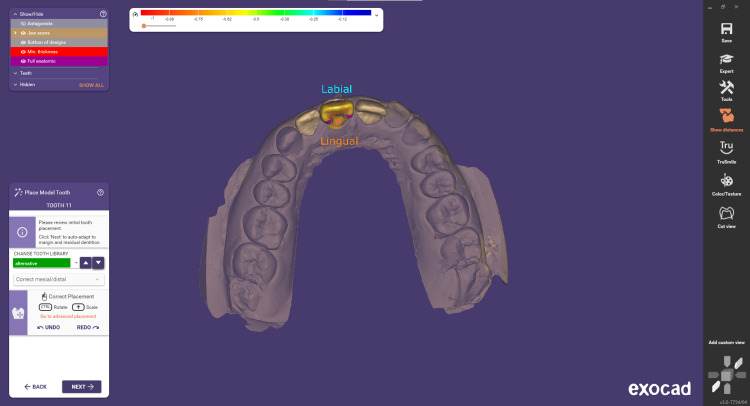
Tooth placement from the Exocad Libraries in occlusal view

**Figure 6:**
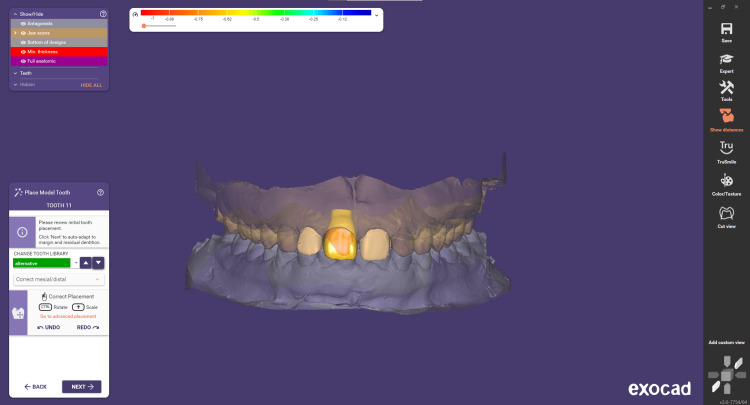
Tooth placement from the Exocad Libraries in frontal view

Step 4 involves adjusting the cement gap. This step focuses on designing the intaglio surface of the crown or inlay, which is the portion that will be in direct contact with the prepared tooth. It is a critical part of the construction process, as the parameters involved significantly influence the proper fitting of the restoration (Figure 4). The cement gap for CAD/CAM-designed crowns, including e.max single crowns, typically ranges from 30 to 50 microns (μm). Step 5 is correct tooth placement. During this step, you can precisely position the model teeth. Within the Tooth Placement window, you'll find three tabs: Simple, Advanced, and Chain Mode. To switch between tooth libraries, use the library drop-down. You can easily navigate through the available libraries by clicking the arrow button, facilitating a streamlined process within the software (Figures 5-6).

**Figure 7:**
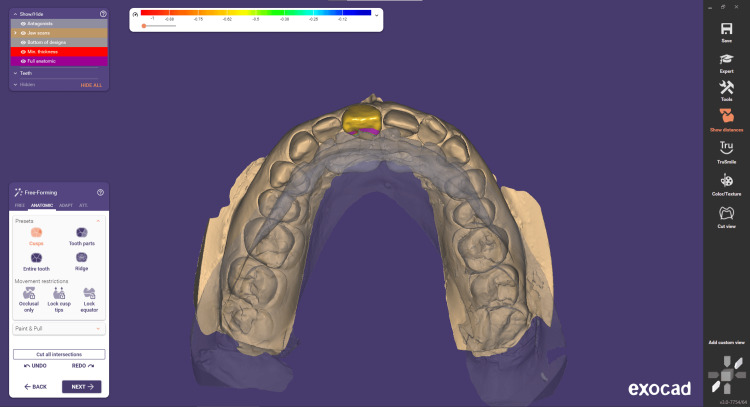
Free-forming step used in order to refine the restoration shape and structure

Step 6 involves free-forming. The "Free Forming" window allows to refine restoration shape and structure with a virtual wax knife, ensuring optimal functionality and aesthetics. Anatomic adjustments mimic the natural tooth morphology for seamless integration. Attachment management streamlines customization, visualizing contacts and occlusion to ensure proper fit and patient comfort. Overall, it offers precise, versatile, and data-driven tools for optimal dental restorations (Figure 7).

**Figure 8:**
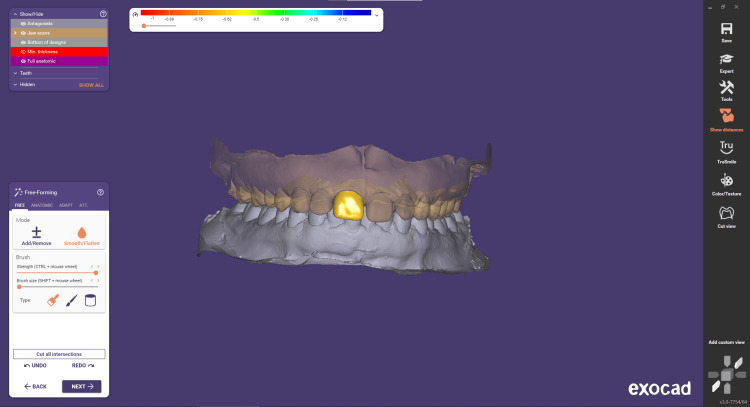
Merging the restoration

**Figure 9:**
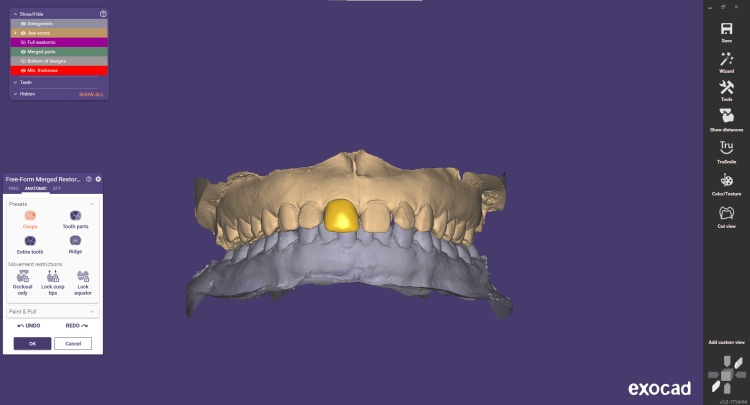
Final anatomic restoration design

**Figure 10:**
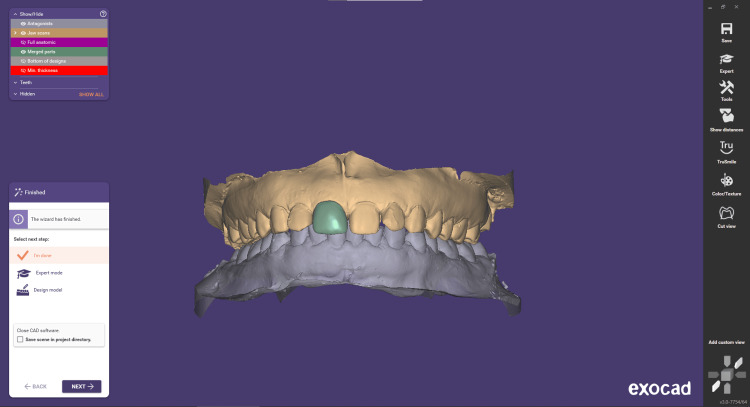
Final anatomic restoration saving the data to STL format

Step 7 involves merging and saving the restoration. During this step, all individual elements designed, including copings, connectors, etc., are combined or merged into one or more meshes suitable for milling or laser melting. Each physical element planned for production results in the creation of one mesh. These meshes are then saved in an STL format, with a cad.stl file extension. Additionally, detailed information such as preparation margin, insertion axes, and other relevant data is written into the .constructionInfo file. This file contains supplementary information that will support CAM applications, ensuring precise fabrication based on the design specifications (Figures 8-10).

**Figure 11:**
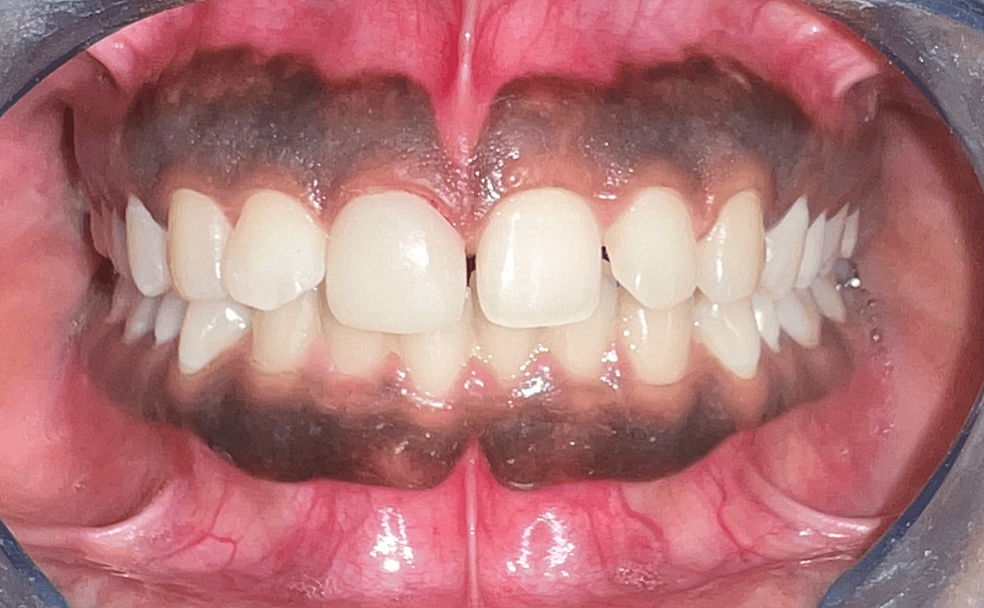
Post cementation of the crown in a one-year follow-up

**Figure 12:**
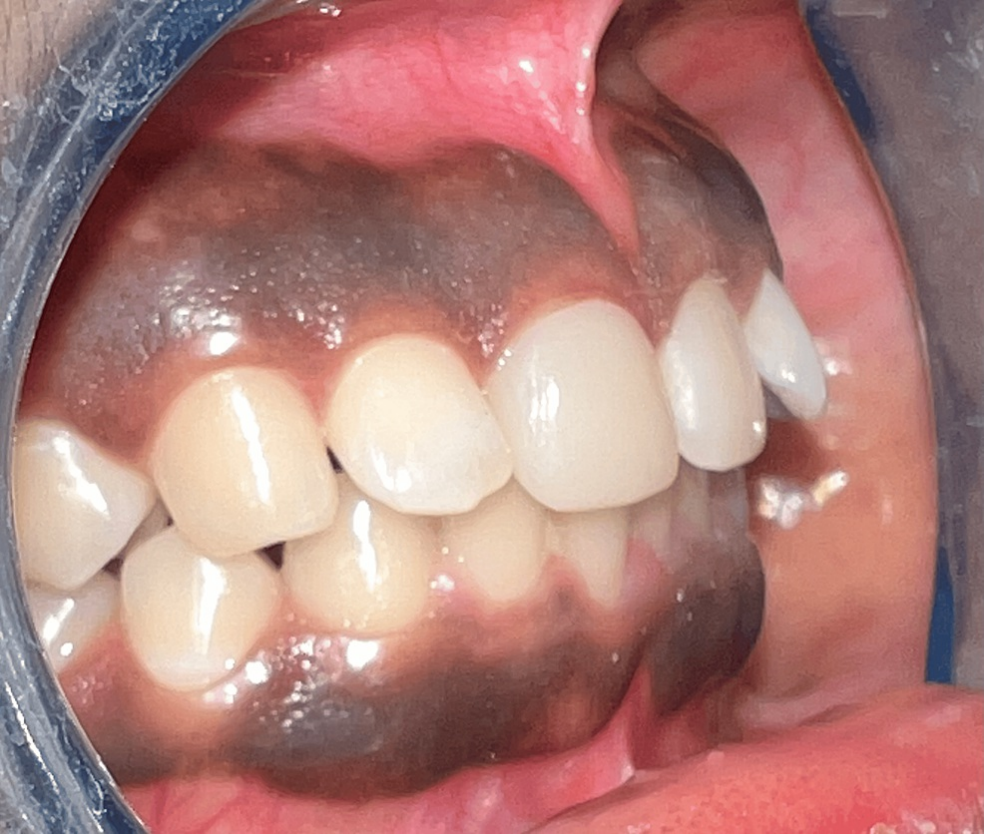
Post cementation of the crown (side view)

The designed crown was transferred to the milling software, and prefabricated high-translucent blocks, specifically e.max, were milled in the in-Lab CEREC MC XL milling machine to be manufactured through the subtractive method to receive a crown for tooth #11. Lithium disilicate was chosen for its high strength and lifelike optical properties, guaranteeing both durability and aesthetic appeal for the restoration. The crown was verified intraorally over prepared tooth #11 to assess marginal fit, contour, and aesthetic harmony. After confirming snug fit of the prosthesis, it was retrieved, cleansed, and air-dried in preparation for the final placement. The crown was seated using the Multilink Automix luting composite, suitable for various restorations. Before applying, the primer solution (Multilink A/B) was manipulated and applied to the preparation enamel and dentine. The luting composite was then applied to the inner surfaces of the crown and carefully seated on tooth #11. Excess cement was removed, and the crown was cured with a light-emitting diode (LED) curing light. Finally, interproximal spaces were flossed to ensure thorough cement removal. The final outcome and one-year follow-up images were taken (Figures 11-12).

## Discussion

Chairside Economical Restoration of Esthetic Ceramics (CEREC, Sirona) represents an innovative CAD/CAM system that addresses the concerns of dental professionals regarding traditional setup challenges. Notably, it separates the milling chamber from the image capture and design hardware, enabling simultaneous design and milling of restorations [7]. With enhanced speed and memory capabilities, CEREC 3D design software offers users a familiar experience akin to evaluating traditional stone models when viewing tooth designs [8, 9].

The CEREC MC XL system is a highly efficient, precise chairside, and in-lab CAD/CAM milling system known for its simplicity and speed. It can process single-crown prostheses and multi-unit FPDs in three to 14 minutes, all within a single appointment. With a margin of error within the range of ± 25 µ, exhibiting both precision and accuracy and a milling resolution of 7.5 µ, it produces improved marginal fit and polished restoration. Additional features such as automatic software installation, easy-to-use display guides, network connectivity, and a user-friendly milling chamber setup make it a versatile and user-friendly system for dental professionals [10].

The innovative pressed ceramic (IPS) e.max CAD (lithium disilicate glass-ceramic by Ivoclar Vivadent) comprises 70% needlelike crystals within a glassy matrix, marking a significant advancement over previous ceramic materials [11]. It exhibits impressive strength values ranging between 360 MPa (press) and 400 MPa (CAD), offering durability and reliability for dental restorations [11-13].

IPS e.max also boasts true-to-life-like optical qualities, allowing dentists to craft highly aesthetic and natural-looking restorations across various cases [14]. This versatile material is suitable for anterior restorations, including veneers (0.3-0.7 mm), minimally invasive inlays and onlays, partial crowns and full contoured crowns, implant superstructures, three-unit bridges for anterior/premolar (press only), and zirconium-oxide-supported IPS e.max CAD only. Its adaptability makes it an ideal choice for a wide range of dental applications, ensuring both aesthetic appeal and functionality [15].

## Conclusions

CAD/CAM technology proved highly beneficial in this case for several reasons. Firstly, it facilitated the efficient and precise fabrication of a lithium disilicate crown for tooth #11 using the Exocad software system, allowing for a single-appointment procedure. Secondly, CAD/CAM enabled intraoral scanning, designing, and milling of the crown, eliminating the need for traditional laboratory work, thus saving time and enhancing patient convenience. Additionally, CAD/CAM provided accurate and lifelike restoration contours, ensuring optimal fit, function, and aesthetics. Lastly, the technology allowed for immediate chairside adjustments and finalization of the restoration, resulting in a highly satisfactory outcome for the patient.
